# Supplementary material for: How Do Pharmacists Distribute Their Work Time during a Clinical Intervention Trial?—A Time and Motion Study
Source: Pharmacy (Basel). 2024 Jul 9;12(4):106. doi: 10.3390/pharmacy12040106 (PMC11270314; doi:10.3390/pharmacy12040106)
Supplement: Supplementary file 1 [file pharmacy-12-00106-s001.zip › pharmacy-3067463-supplementary.pdf]

Supplementary File S1: The final version of the WOMBAT (Work Observation Method By Activity Timing) tool

The final version of the WOMBAT outline consisted of five dimensions, as illustrated in Figure S1 (in beige). Data were collected using an iPad® Mini with the WOMBAT software version 3.0 installed (see Figure S1 for a screenshot). The WOMBAT software allows for the collection of time-stamped observational data, recording the exact time from when you start/press the current "what" task until a new task is started or interrupted. It provides a quick and easy transition between task categories. * Indicates mandatory dimensions. The currently running task is automatically marked in green, and on the left panel, the previously time-stamped tasks and currently running task are shown.

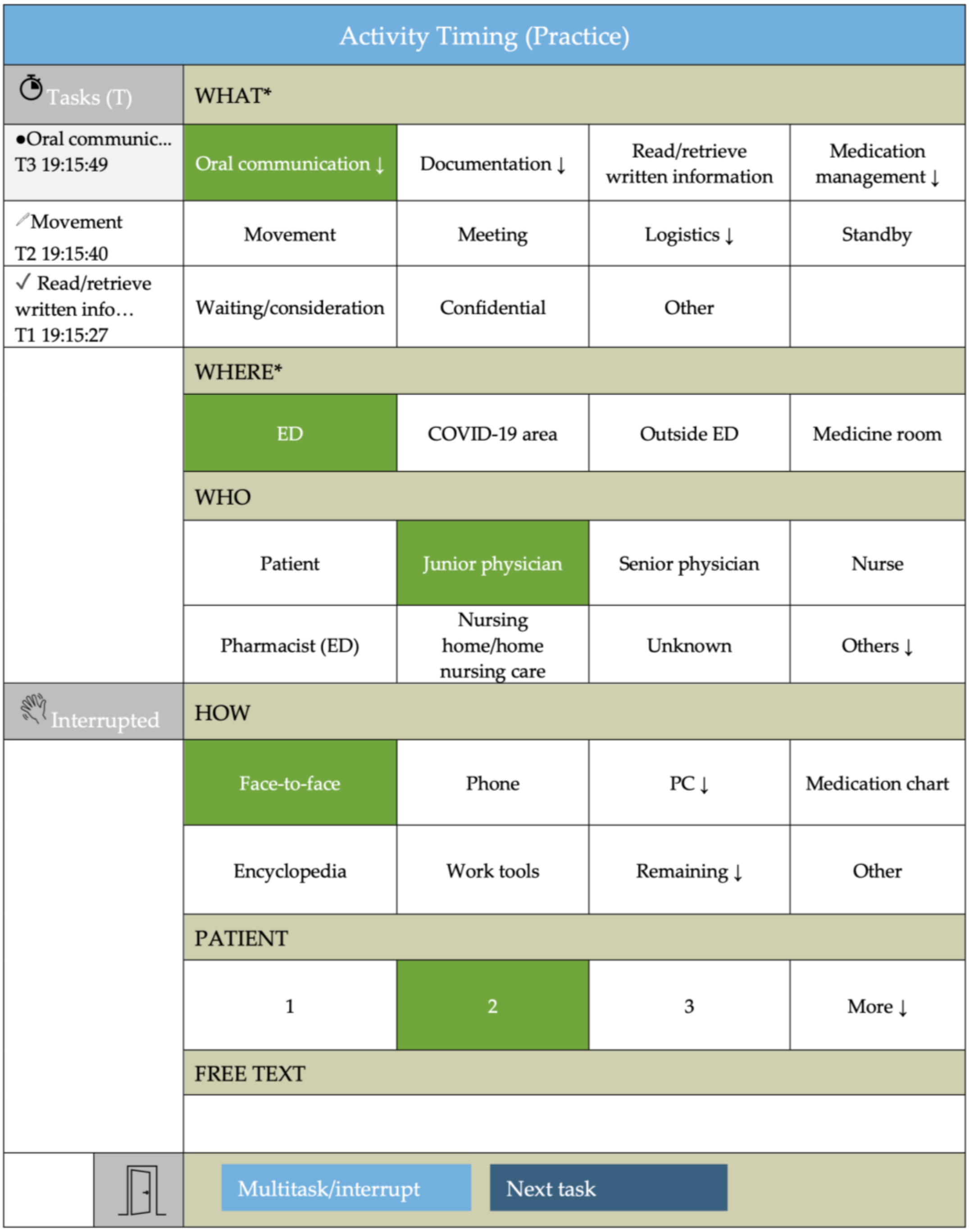

Figure S1. Illustration of the WOMBAT software with the five dimensions and associated categories.

1. WHAT

Describes which task that is performed by the observed pharmacist. This dimension was mandatory. Definitions and examples of the categories and sub-categories in this dimension are provided in Supplementary File S2. The down arrow indicates that there are sub-categories chosen from a drop-down menu, consisting of the following:

- Oral communication ↓
- Retrieve medication-related information
- Communication about medications
- Work-/patient-related
- Documentation ↓
- Medication-related
- Non-medication-related
- Logistics ↓
- Other
- Medication-related
- Planning

2. WHERE

Describes where the observed pharmacist was when conducting the recorded task. This dimension was mandatory, with only one option possible to register.

3. WHO

Describes with whom (if anyone) the observed pharmacist performed the task. This dimension was not mandatory, as many work tasks are conducted without interaction with others. Multiple options are possible to register, e.g., oral communication with a nurse and a senior physician. The down arrow indicates that there are other choices available from a drop-down menu:

Others ↓
- Specialist physician
- Healthcare personnel
- Medical student
- Next-of-kin
- Outside hospital

4. HOW

Describes, practically, how the observed pharmacist conducted the task. This dimension was not mandatory, as some tasks do not require explaining how they are performed, e.g., movement. Multiple options were possible to register, e.g., reading and retrieving information from the prescription intermediary and the Summary Care Record at the same time. The down arrow indicates that there are other choices available from a drop-down menu:

PC ↓
- Electronic Health Record
- Medication module in Electronic Health Record
- Prescription intermediary
- Summary Care Record
- Interaction information screen
- Electronic chart
- Other on PC
- Voice recorder

5. PATIENT

Every patient who was treated, communicated with or about during the day of the observation was registered with a unique number. The drop-down menu consisted of

numbers up to 30, including “x”, which was used if the patient was unknown to the observer. This dimension was not mandatory as not all the conducted tasks involve patients, e.g., being on standby. Only one option was possible to register.

More ↓

- 4
- 5
- … up to 30
- X (unknown patient)

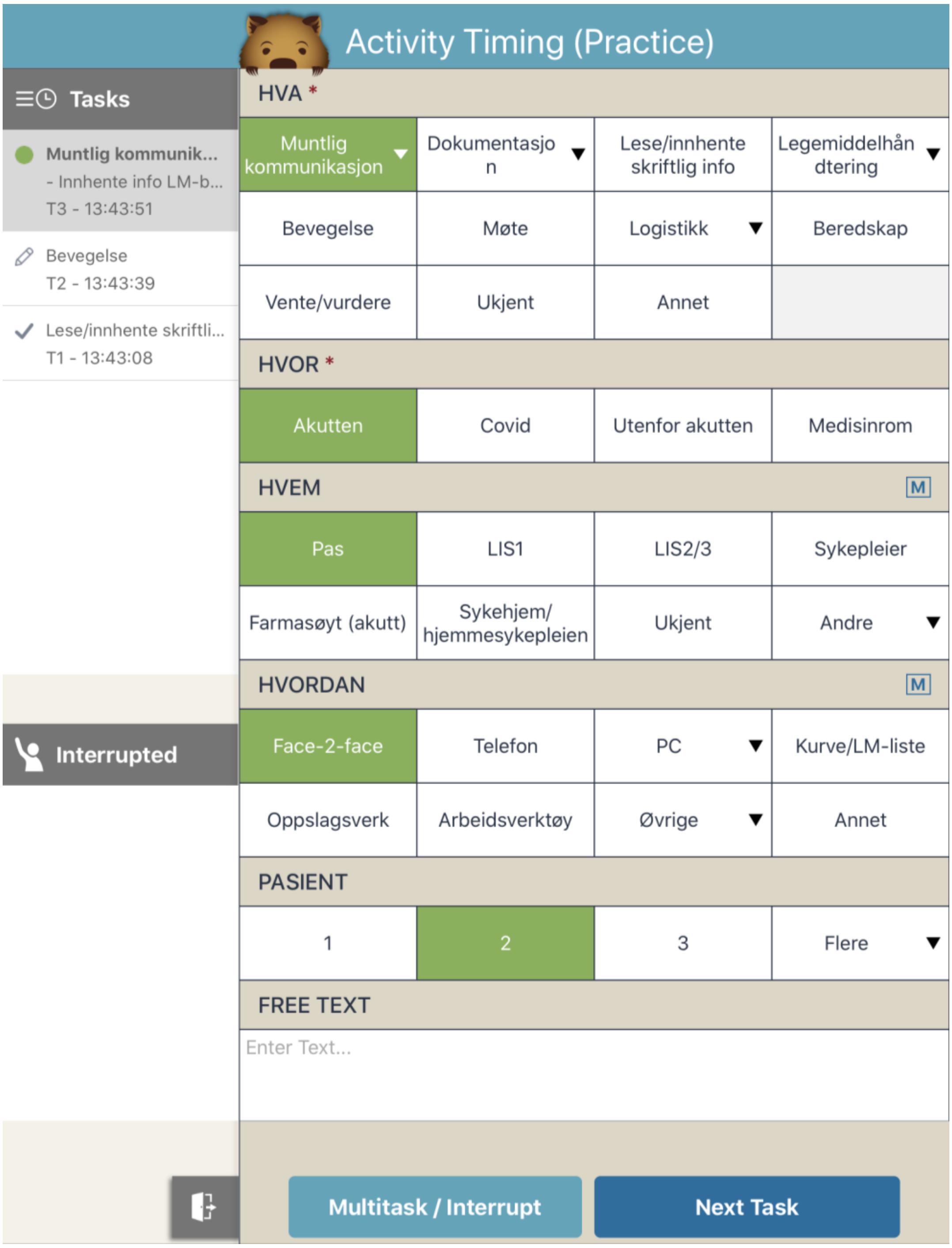

Figure S2: Screenshot of the actual WOMBAT software used in the study (with the Norwegian language).

Supplementary File S2. Definitions of the WHAT dimensions in the WOMBAT (Work Observation Method By Activity Timing) tool

Table S1. The "What" dimension's categories, sub-categories, definitions, and examples.

WHAT CATEGORY	WHAT SUB-CATEGORY	DEFINITION	EXAMPLE
**Oral communication**	Retrieve medication-related information	Retrieving information about a patient's medication use	Talks to, e.g., the patient or home care nurse about a patient's home medications
	Communication about medications	Communication about medications between healthcare personnel or when medication-related information provided to the patient/relatives	Physician and pharmacist discuss a patient's medication list
	Work-/patient-related	Communication with or about the patient with healthcare personnel Work-related communication between colleagues. Not medication-related communication.	Nurse informs pharmacist about triage results
**Read/retrieve written information**	-	Reading in Electronic Health Record or encyclopedia	Read previous discharge notes in Electronic Health Record or checking blood test results
**Documentation**	Medication-related	Documenting a patient's medications	Writing medical chart
	Non-medication-related	Documenting patient history or blood tests	Writes about a patient's previous medical history in Electronic Health Record
**Movement**	-	Movement from one place to another, within the ED or between departments	Moving from the break room to the patient room
**Medication management**	Pharmaceutica calculations	Calculation of dosage, strength, or quantity of a medication	Calculation of dosage, strength, or quantity of a medication
**Waiting/consideration**	-	Pharmacist is not directly active in the work task, could be, e.g., thinking, considering or waiting for test results	A pharmacist waiting for results of the urine sample
**Logistics**	Other	Assist with moving patients, changing bed linens, preparing rooms	A nurse cleans a patient room
	Medication-related	Retrieve medications at the pharmacy/department, tidy up medications in the medicine room	Checking the expiration date of the medications available in the medicine room

	Non-medication-related	Planning, assessment and prioritization of patients	Attending patients with longer medication lists rather than patients without any medications
**Standby**	-	Time spent not doing any specific work tasks	Lunch/toilet break, inactive/available time (e.g., no patients in the ED).
**Meeting**	-	Staff meeting, morning meeting, internal teaching/education	Morning meeting where they summarize the previous 24 h
**Confidential**	-	Not observable work tasks	Tasks that were conducted in private, without the presence of an observer, due to their sensitive nature and the need for discretion, or due to prevention of infectious diseases.
**Other**	-	Work tasks inapplicable with the other categories.	A pharmacist washing hands, completely independent on some of the other work tasks
